# Astragalus Polysaccharide Attenuates Breast Cancer Progression by Regulating METTL3-Mediated MAL2 m^6^A Modification

**DOI:** 10.4014/jmb.2510.10015

**Published:** 2026-01-22

**Authors:** Youting Hu, Kongjun Zhu, Jing Zhang, Jianguo Zhao

**Affiliations:** Department of Breast and Thyroid Surgery, Wuhan Hospital of Traditional Chinese and Western Medicine, Wuhan 430000, Hubei, P.R. China

**Keywords:** *Astragalus* polysaccharide, Breast cancer, MAL2, METTL3, m^6^A modification

## Abstract

Astragalus polysaccharide (APS) has recently emerged as a potent antitumor agent, however its impact on breast cancer (BC) remains inadequately understood. The current research aimed to examine the regulatory mechanism of APS in the pathogenesis of BC examining its influence on N^6^-methyladenosine (m^6^A) modification of MAL2. The effect of APS on the malignant phenotypes of BC was assessed by CCK8, EdU, transwell and tumor xenograft model assays. The differentially expressed genes (DEGs) in BC were identified by GEPIA-BC database, and their expression levels were determined by qRT-PCR in the BC cells. The role of MAL2 in BC malignancy was examined by EdU and transwell assays. Furthermore, bioinformatics analysis was first employed to explore the m^6^A modification site of MAL2 mediated by METTL3, which was then validated through MeRIP, western blotting, and qRT-PCR assays. APS was found to significantly reduce the cell proliferation, migration, as well as invasion of MCF-7 (IC50: 1014 μg/ml) and MDA-MB-231 (IC50: 685 μg/ml) cell lines. Additionally, it effectively suppressed tumor growth *in vivo*. The bioinformatics analysis revealed that among the five DEGs, MAL2 was significantly downregulated upon APS treatment both BC cell lines. Furthermore, the overexpression of MAL2 partially reversed the anti-tumor effects of APS. Notably, METTL3 modulates the m6A modification of MAL2 to regulate tumorigenesis in BC. APS prevents BC progression in association with reduced METTL3 expression and altered m^6^A modification of MAL2, suggesting that MAL2 may represent a potential therapeutic target to enhance the efficacy of APS.

## Introduction

Breast cancer (BC) is the most widespread malignancy that affects females globally, with more than 2.3 million cases and nearly 665,000 deaths reported alone in the year 2022 [1]. BC is a complex disease driven by both genetic and environmental factors, with current treatment options including surgery, chemoradiotherapy, endocrine therapy, and targeted therapy widely used in clinical practice [2]. Nonetheless, while various chemo preventive drugs are effectively used to treat BC, challenges such as chemoresistance and metastasis persist in certain patients, particularly in triple-negative BC, often resulting in a poor prognosis [3]. Hence, there is an immediate need to develop safer and more effective natural drugs for BC treatment and to investigate their underlying mechanisms of action.

Recently, traditional Chinese herbal medicine, particularly plant extracts, have gained the attention of the researchers as they are abundant in bioactive compounds and demonstrate promising antitumor effects [4, 5]. Their low toxicity and minimal side effects make them appealing as both therapeutic agents and dietary components in cancer care. Among plant extracts used in Chinese herbal medicine, *Astragalus* polysaccharide (APS) from *Astragalus membranaceus* have been highlighted for their potential therapeutic effects in BC [6, 7]. A previous report has shown that APS suppressed the growth of BC cells by enhancing the immune response [8]. Another study established that APS intervenes with invasion, proliferation, and apoptosis of triple-negative BC cells via the PIK3CG/AKT/BCL2 pathway [9]. Furthermore, APS reduced the proliferation, and Epithelial-Mesenchymal Transition-driven migration and invasion of BC cells by blocking the Wnt/β-catenin pathway [10]. Nonetheless, the potential targets and possible mechanisms behind the anti-tumor activity of APS in BC remains unclear.

The progression of BC involves the interplay of multiple genes and changes in gene expression profiles have become a key focus in BC research with numerous differentially expressed genes (DEGs) identified in patients [11]. Abnormal gene expression is known to drive malignancy progression, and targeting DEGs has emerged as a promising strategy in various antitumor clinical studies [12]. Using the GEPIA-BC database, we identified five DEGs and selected MAL2 for further study due to its significant downregulation upon APS treatment in both BC cell lines. MAL2 (Myelin and lymphocyte protein 2 or T-cell differentiation protein 2) is a four-pass transmembrane protein consisting of 176 amino acids, with recent reports highlighting its role as a crucial regulator in various cancers, including BC [13]. Increased expression of MAL2 has been observed in several cancers, such as ovarian, papillary thyroid, pancreatic, and non-small cell lung cancers, and is often linked with poor patient prognosis [14-17]. MAL2 acts as an oncogene in BC, and has been shown to be overexpressed in BC tissues and cells [18, 19]. Despite MAL2’s potential as both a therapeutic target and a prognostic biomarker for BC, no specific drugs have been identified to target it.

Currently, N^6^-methyladenosine (m^6^A) is a widespread RNA modification that affects RNA expression, stability, and processing in various biological contexts [20]. This modification is carried out by a methyltransferase complex, with Methyltransferase-like 3 acting as the key catalytic subunit responsible for methylating RNA molecules [21]. Given METTL3's crucial role in RNA modification, it is important to explore its potential impact on cancer-related processes. Recently, plant extracts have been investigated for their potential to modulate m^6^A methylation [22-24]. However, the m^6^A regulation of APS in BC remains largely unexplored. Therefore, the aim of this work was to examine the potential protective effects of APS in BC and determine whether these effects were mediated through the modulation of m^6^A RNA methylation.

## Materials and Methods

### Cell Culture and Transfection

Human normal breast epithelial cell line MCF-10A (cat. no. CL-0525), and two BC cell lines including MCF-7 (cat. no. CL-0149) and MDA-MB-231 (cat. no. CL-0150) were purchased from Procell, China. The MCF-10A cells were cultured in CM-0525 medium (Procell), and the BC cell lines were cultured in DMEM medium (Procell) containing 10% FBS (Procell) and were incubated under the condition of 5% CO_2_ and 37°C.

The overexpression vectors of MAL2 (pcDNA3.1-MAL2) and METTL3 (pcDNA3.1-METTL3) were constructed by RiboBio, China using the pcDNA3.1 vector, while the empty vector served as a negative control (pcDNA3.1). Additionally, Lipofectamine 3000 (Thermo Fisher Scientific, USA) was used for cell transfection and the efficiency of transfection was checked at 48 h post-transfection via qRT-PCR.

For APS (Absin, China) treatment, MCF-7 and MDA-MB-231 cells were subjected to increasing concentrations of APS that are as follows: 0, 25, 50, 100, 200, 400, 800, and 1,600 μg/ml. The viability of the cells with APS treatment was evaluated via a CCK-8 assay and then IC50 was determined. The cells subjected to the DMSO treatment were taken as the control.

### Extraction of RNA and Quantitative Real Time PCR (qRT-PCR)

The total RNA was extracted from both the BC cell lines (MCF-7 and MDA-MB-231) via Trizol method by utilizing the Trizol reagent (Invitrogen, USA) and quantified with the help of a NanoDrop spectrophotometer (Thermo Fisher Scientific). Thereafter, reverse transcription reactions were done by means of a PrimeScript RT Reagent Kit (Takara Biotechnology Co., Ltd., China). Then, using SYBR Green qPCR Kit (Takara Biotechnology), qRT-PCR was carried out. The quantitative analysis of the data was done by adopting 2^−ΔΔCt^ method with GAPDH as the internal control. The primer sequences are shown in Table 1.

### Cell Counting Kit-8 (CCK-8) Assay

After undergoing a 48-h APS treatment with different concentrations, the cells (2000 cells/well) were plated in 96-well plates. Afterward, CCK-8 solution (10 μL, Beyotime, China) was dispensed into each well and a 4-h incubation was done at 37°C. Finally, absorbance was recorded at 450 nm by utilizing a microplate reader (Thermo Fisher Scientific).

### EdU Assay

The Cell-Light EdU Apollo643 *In Vitro* Kit (RiboBio, China) was employed to carry out EdU assay. Briefly, BC cells (5,000 cells/well) were seeded in 96-well plates and left to incubate for 24 h. Following this, the cells were exposed to EdU reagent (10 μM) for 4 h, fixed and permeabilized using paraformaldehyde (4%) and Triton X-100 (0.5%), respectively. They were then stained with 1× Apollo reagent for 30 min. The nuclei were labeled with 1× DAPI, and cells were observed under a fluorescence microscope.

### Transwell Migration/Invasion Assays

Migration and invasion assays were done by employing Millipore transwell inserts in a 24-well plate. The invasion assay involved 8% Matrigel (BD Biosciences, USA) precoated upper transwell chamber, whereas the migration assay used uncoated membranes. In the bottom chamber, DMEM (600 μl) supplemented with 20% FBS was added, and BC cells were seeded in serum free DMEM (200 μl) in the upper chamber. After a 24-h incubation at 37°C, the migrated and invaded cells were fixed with paraformaldehyde (4%), stained with crystal violet (0.1%), and visualized using microscopy.

### Tumor Xnograft Model

The ethics committee of Wuhan Hospital of Traditional Chinese And Western Medicine approved the animal experiments (approval number: 2024015). Twelve male nude mice (6 to 8 weeks old) were obtained from Vital River, China and randomly assigned to four experimental groups, with three mice in each group. Then, 1 × 10^6^ MCF-7 and MDA-MB-231 cells, with or without APS treatment at 1014 μg/ml and 685 μg/ml respectively, were subcutaneously implanted into the right flank of the mice. Tumor volume (½length × width^2^) was recorded every 7 days, and the mice were euthanized after 5 weeks.

### Methylated RNA Immunoprecipitation (MeRIP) Assay

This assay was accomplished using the riboMeRIP m^6^A Transcriptome Profiling Kit (RiboBio). Following the guided instructions, 100 μg RNA from BC cells with pcDNA3.1-METTL3 or pcDNA3.1 transfection were fragmented into 100–150 bp fragments. The fragment RNA was then treated overnight with beads coated in anti-m^6^A or IgG overnight. After washing beads with RIP wash buffer, the m^6^A enrichment of MAL2 was detected by qRT-PCR.

### Western Blotting

The BC cells were lysed in RIPA lysis buffer (Beyotime) and the amount of protein was evaluated via a Pierce BCA Protein Assay Kit (Thermo Fisher Scientific). The proteins were separated by SDS-PAGE, and then transferred to PVDF membranes. Thereafter, these membranes were blocked with skim-milk (5%) for 2 h at room temperature (RT), and an overnight (O/N) incubation was done at 4°C with the various primary antibodies, which are: anti-METTL3 (abs118443, Absin), anti-MAL2 (AP55313SU-N, OriGene, USA), and anti-GAPDH (abs132004, Absin). Next morning, the membranes were incubated for 1 h at RT with the HRP-labelled secondary antibody raised in rabbit (Absin) and the protein bands were visualized with BeyoECL Plus (P0018S, Beyotime).

### Statistical Analysis

GraphPad Prism 8.0 (GraphPad, USA) was utilized in the analysis of the data. The data were all represented by their mean ± SD. T-test and analysis of variance (ANOVA) followed by Tukey post-hoc test were applied accordingly to compare the differences among two and multiple groups, respectively. *P* < 0.05 is indicative of a significant difference.

## Results

### APS Effectively Suppressed the Malignant Phenotypes of BC Cells

**Fig. 1:**
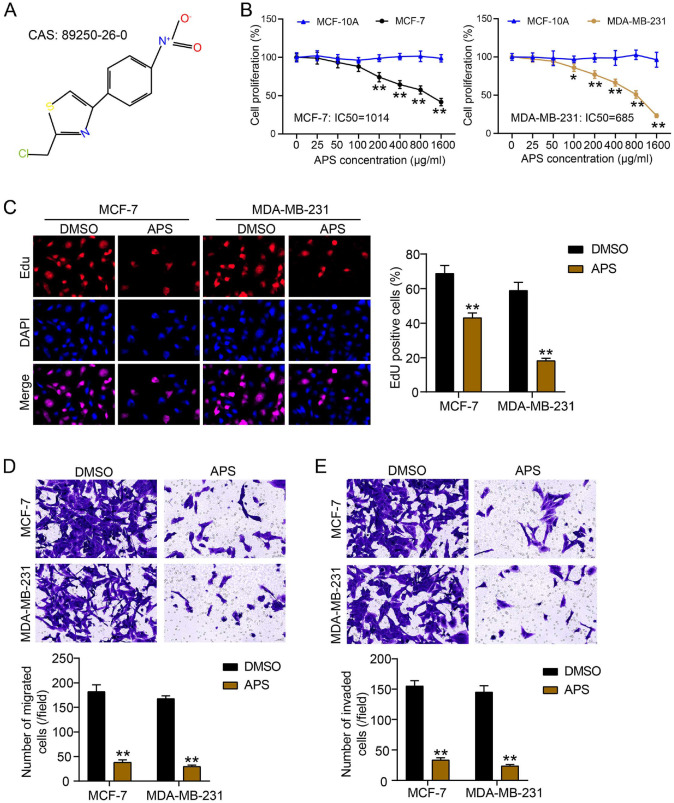
APS inhibit the malignant properties of BC cells. (**A**) The structure of APS in PubChem. (**B**) CCK8 assessed the viability of BC cells with different concentration of APS (0~1,600 μg/ml) in BC cell lines (MCF-7 and MDA-MB-231) and human normal breast epithelial cell line MCF-10A. [*P < 0.05, **P < 0.001 vs. 0 μg/ml APS]. (**C**) The effect of APS at concentrations of 1,014 μg/ml and 685 μg/ml on the proliferation of MCF-7 and MDA-MB-231 cells, respectively, was evaluated using the EdU assay. (**D**) The effect of APS at concentrations of 1014 μg/ml and 685 μg/ml on the migration of MCF-7 and MDA-MB-231 cells, respectively, was assessed by Transwell migration assay. (**E**) The effect of APS at concentrations of 1014 μg/ml and 685 μg/ml on MCF-7 and MDA-MB-231 cell invasion, respectively, was analyzed using a Transwell invasion assay. [C to E: **P < 0.001 vs. DMSO]. Data are presented as mean ± SD of three independent biological replicates (*n* = 3).

The structure of APS from PubChem (https://pubchem.ncbi.nlm.nih.gov/) is shown in Fig. 1A. We first evaluated the effect of different APS concentrations on the viability of BC cells (MCF-7 and MDA-MB-231) and human normal breast epithelial cells MCF-10A via CCK8 assay. The results demonstrated that 1,014 μg/ml and 685 μg/ml APS induced an approximately 50% decline in viability among MCF-7 and MDA-MB-231 cells, respectively (Fig. 1B). However, APS could not affect MCF-10A cell viability, indicating that APS did not exhibit cytotoxicity in normal cells. Based on these results, 1,014 μg/ml and 685 μg/ml concentrations of APS were utilized for the subsequent experiments. The EdU assay revealed that APS significantly reduced BC cell proliferation, as indicated by a reduction in the percentage of EdU-positive cells after APS treatment (Fig. 1C). Furthermore, the transwell assays uncovered that APS notably lowered the number of migratory and invasive BC cells, suggesting a broad effect of APS on BC cell migration and invasion (Fig. 1D and 1E). Collectively, these findings illustrated that APS significantly reduced the malignant behavior of BC cells *in vitro*.

### APS Restricted the Growth of BC Cells in a Mouse Subcutaneous Xenograft Model

**Fig. 2:**
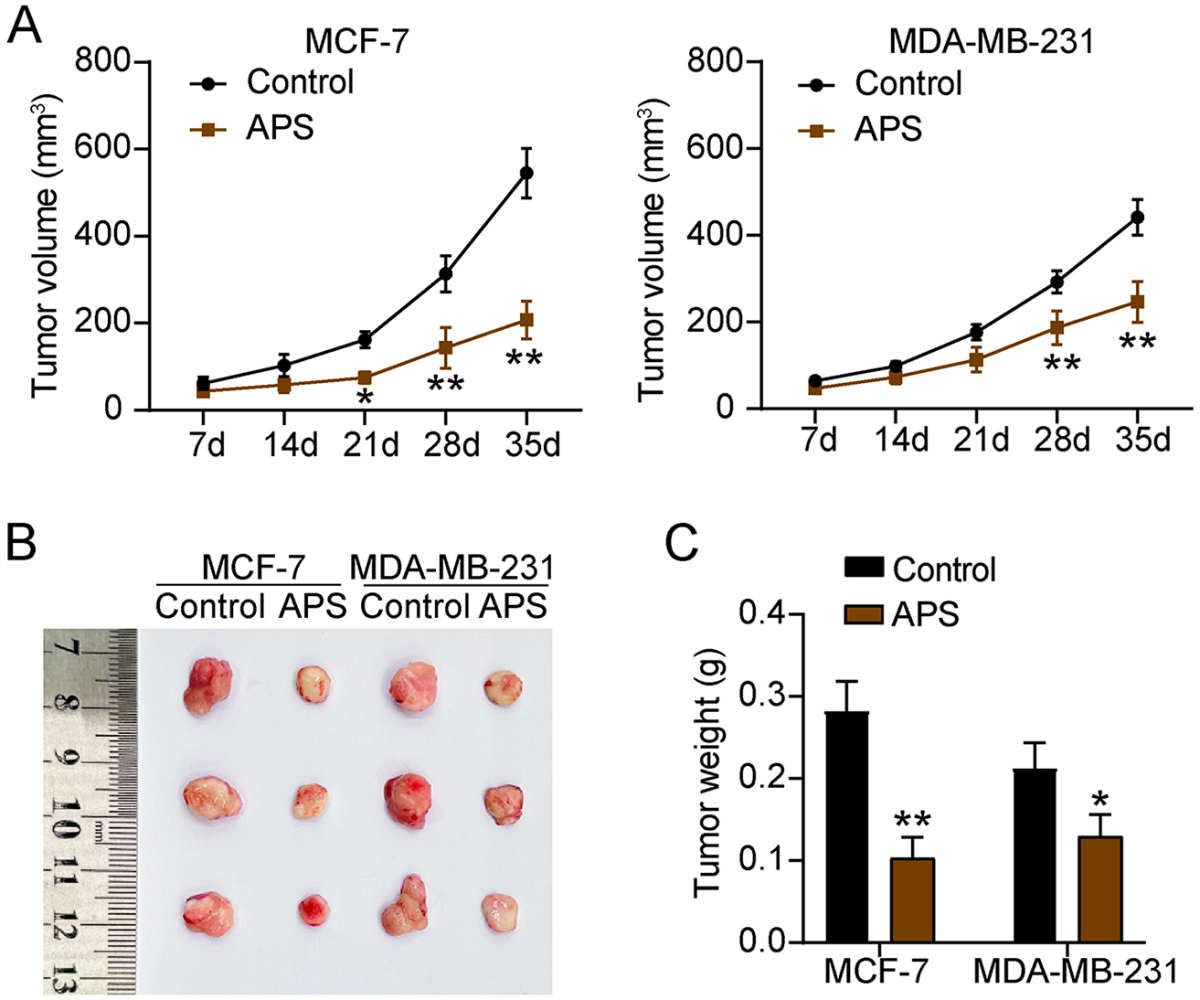
APS inhibit the growth of BC in a mouse subcutaneous xenograft model. MCF-7 and MDA-MB-231 cells, with or without APS treatment at 1,014 μg/ml and 685 μg/ml respectively, were subcutaneously implanted into the right flank of the mice to establish the xenograft model. (**A**) The graph showing tumor sizes measured over a 5-week period, highlighting changes in tumor volume over time. (**B**) The images of the tumors extracted from the mice, which visually captures the effects of the APS treatment. (**C**) A bar chart illustrating the differences in the weights of harvested tumors after 5 weeks [*P < 0.05**P < 0.001 vs. Control]. Data are presented as mean ± SD (*n* = 3).

To determine whether the effects of APS on tumor growth *in vivo*, a mouse subcutaneous xenograft model was established using MCF-7 and MDA-MB-231 cells. The tumor growth restriction effect became apparent after 21 days of APS treatment, as demonstrated by a reduction in tumor volume (Fig. 2A). Subsequently, after 35 days of APS treatment, the harvested tumors exhibited a clear reduction in both size and weight (Fig. 2B and 2C). These findings suggested that APS effectively suppressed BC tumor growth *in vivo*, supporting its anti-tumor efficacy in BC.

### APS Markedly Downregulated MAL2 Expression in BC Cells

**Fig. 3:**
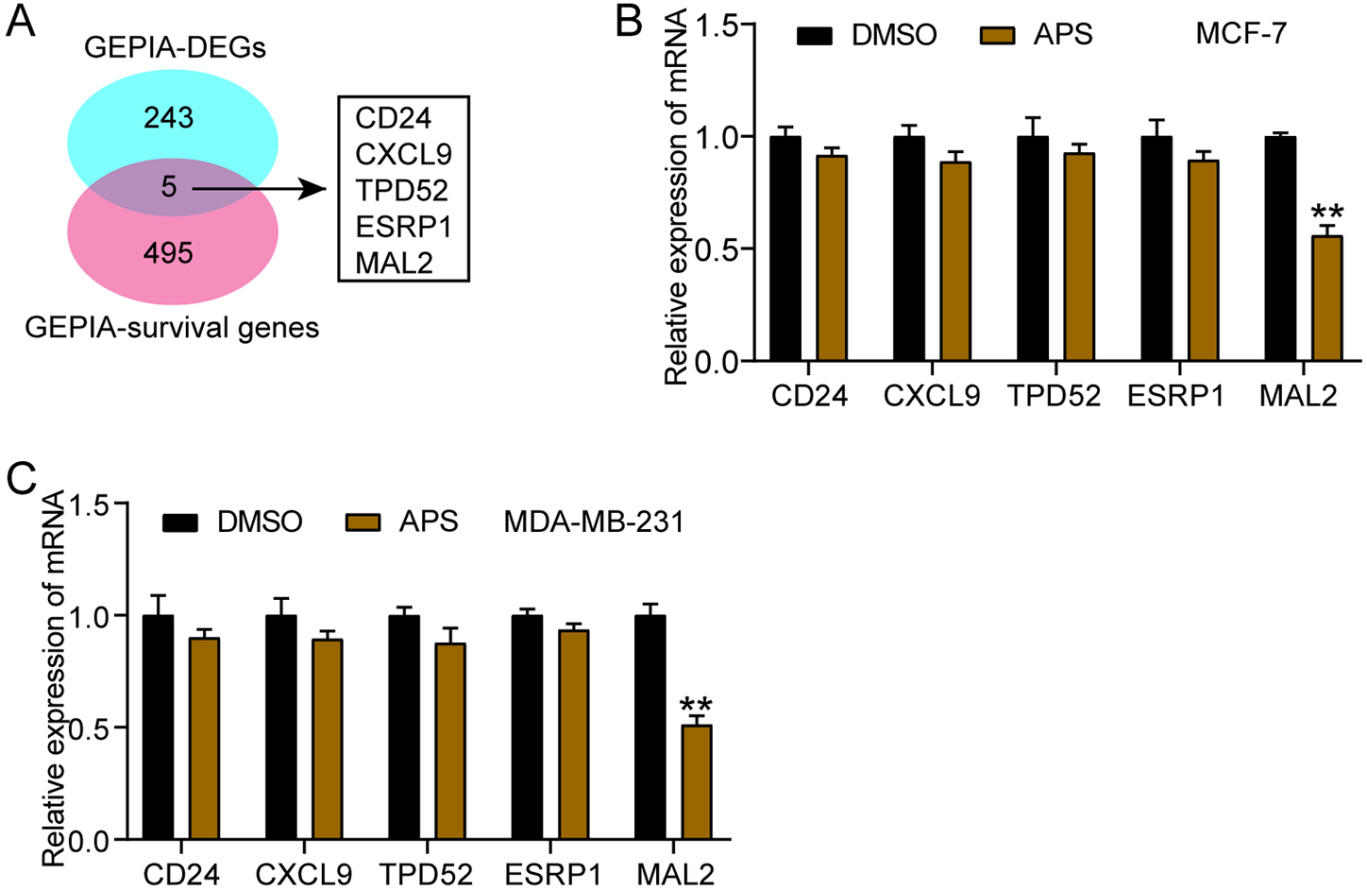
APS significantly lowers the levels of MAL2 in BC cells. (**A**) Veeny2.1.0 tool demonstrating the overlapped 5 common genes from differentially expressed genes (DEGs) BC and survival genes in GEPIA-BC database. (**B**) The effect of APS treatment at concentrations of 1,014 μg/ml on expression levels of the 5 DEGs in MCF-7 cells was assessed via RT-qPCR. (**C**) The effect of APS treatment concentrations of 685 μg/ml on expression levels of the 5 DEGs in MDA-MB-231 cells was assessed via RT-qPCR. [**P < 0.001 vs. DMSO]. Data are presented as mean ± SD of three independent biological replicates (*n* = 3).

To explore potential molecular targets underlying the antitumor effects of APS, bioinformatic analysis was performed to identify genes associated with both BC progression and patient survival. The Veeny 2.1.0 tool identified five common genes that overlap between differentially expressed genes (DEGs) in BC and survival-related genes in the GEPIA-BC database, namely CD24, CXCL9, TPD52, ESRP1, and MAL2 (Fig. 3A). Interestingly, APS treatment led to a considerable reduction in the relative expression of MAL2 mRNA exclusively in MCF-7 (Fig. 3B) and MDA-MB-231 (Fig. 3C) cells, whereas the other candidate genes were not consistently affected. These findings suggested that MAL2 might represent a key molecular target through which APS exerted its anti-tumor effects in BC.

### MAL2 Overexpression Partially Counteracted the Tumor-Suppressive Effects of APS on BC Cells

**Fig. 4:**
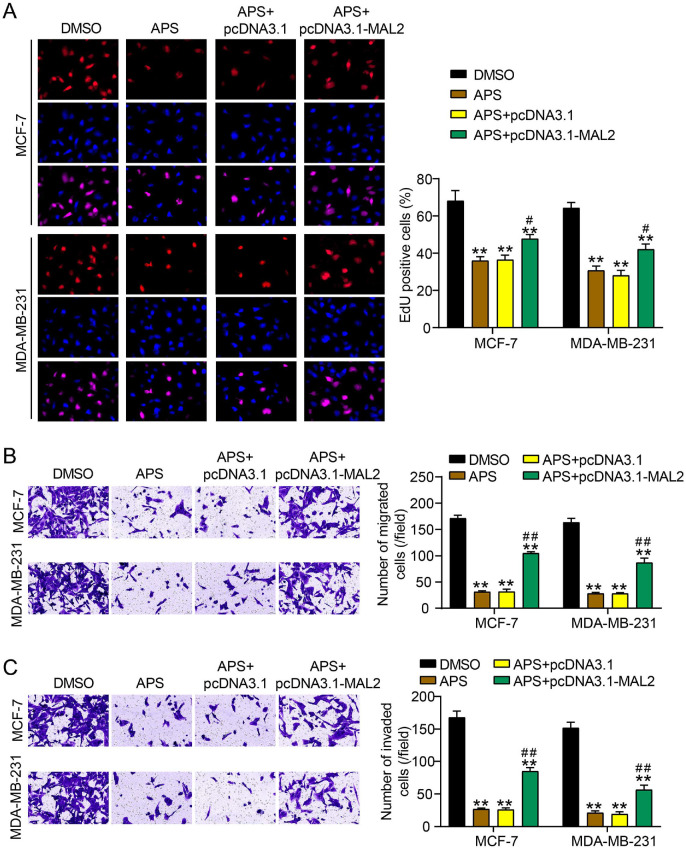
MAL2 overexpression partially reverse the anti-tumor effects of APS on BC cells. (**A**) The cell proliferation was evaluated using the EdU assay to investigate the proliferative capacity of MCF-7 and MDA-MB-231 cells following treatment with APS at concentrations of 1,014 μg/ml and 685 μg/ml, respectively, either alone or in combination with pcDNA3.1-MAL2 transfection. (**B**) The Transwell migration assay was done to investigate the migratory capacity of MCF-7 and MDA-MB-231 cells following treatment with APS at concentrations of 1,014 μg/ml and 685 μg/ml, respectively, either alone or in combination with pcDNA3.1-MAL2 transfection. (**C**) The Transwell invasion assay was performed to assess the invasive potential of MCF-7 and MDA-MB-231 cells after treatment with APS at concentrations of 1,014 μg/ml and 685 μg/ml, respectively, either alone or in combination with pcDNA3.1-MAL2 transfection. [*P < 0.05, **P < 0.001 vs. DMSA; ^#^*P* < 0.05, ^##^*P* < 0.001 vs. APS+pcDNA3.1]. Data are presented as mean ± SD of three independent biological replicates (*n* = 3).

To further verify whether APS targeted MAL2 to inhibit BC cell malignancy, rescue experiments were performed by transfecting pcDNA3.1-MAL2 into APS-treated BC cells. EdU assay demonstrated that MAL2 overexpression partially restored the proliferative capacity of BC cells suppressed by APS treatment (Fig. 4A). Similarly, the decrease in the quantity of migrated and invaded BC cells following APS treatment was significantly reversed by MAL2 overexpression, indicating that APS-induced inhibition of BC cell migration and invasion was attenuated by MAL2 overexpression (Fig. 4B and 4C). The data clearly elucidated that MAL2 overexpression partially reversed the tumor-suppressive effects of APS on BC cells.

### APS Reduced METTL3 Expression and Altered m^6^A Modification of MAL2 in BC Cells

**Fig. 5:**
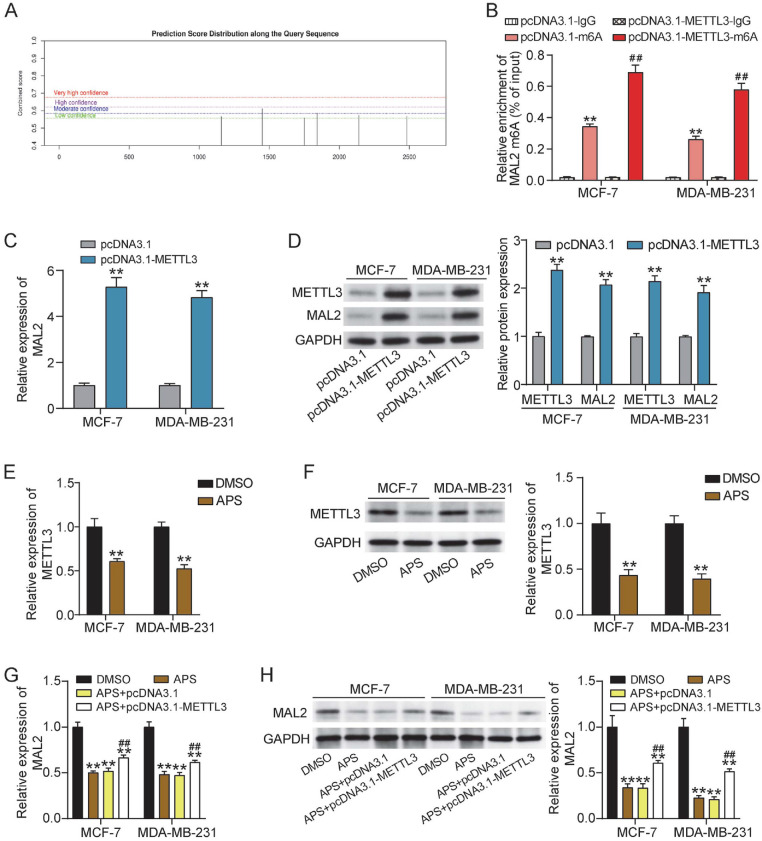
APS reduced METTL3 expression and altered m^6^A modification of MAL2 in BC cells. (**A**) The m^6^A modification sites of MAL2 as assessed by SRAMP (http://www.cuilab.cn/sramp). (**B**) MeRIP assay was carried out to detect the m^6^A enrichment of MAL2 in BC cells with METTL3 overexpression vector (pcDNA3.1-METTL3) transfection. [**P < 0.001 vs. pcDNA3.1-1gG; ^##^*P* < 0.001 vs. pcDNA3.1-METTL3-IgG]. (**C**) The relative expression of MAL2 was analyzed in BC cells with METTL3 overexpression vector (pcDNA3.1-METTL3) transfection by qRT-PCR. [**P < 0.001 vs. pcDNA3.1]. (**D**) The relative protein expression of METTL3 and MAL2 was analyzed by Western blotting in MCF-7 and MDA-MB-231 cells transfected with METTL3 overexpression vector (pcDNA3.1- METTL3). [**P < 0.001 vs. pcDNA3.1]. (**E-F**) The relative expression of METTL3 mRNA and protein was evaluated by qRT-PCR and western blotting in MCF-7 and MDA-MB-231 cells treated with APS at concentrations of 1,014 μg/ml and 685 μg/ml, respectively. [**P < 0.001 vs. DMSO]. (**G-H**) The relative expression of MAL2 mRNA and protein was evaluated by qRT-PCR and western blotting in MCF-7 and MDA-MB-231 cells treated with APS at concentrations of 1,014 μg/ml and 685 μg/ml, respectively, with or without pcDNA-METTL3. [**P < 0.001 vs. DMSO; ^##^*P* < 0.001 vs. APS+pcDNA3.1]. Data are presented as mean ± SD of three independent biological replicates (*n* = 3).

To check whether APS influences RNA m^6^A in BC, we first assessed the m^6^A modification site of MAL2 by SRAMP (Fig. 5A). We then conducted MeRIP-qPCR assay to clarify whether METTL3 could directly mediate the m^6^A modification of MAL2 mRNA in BC cells. The results demonstrated that METTL3 overexpression increased m^6^A enrichment of MAL2 mRNA (Fig. 5B). Furthermore, the qRT-PCR and western blotting analysis illustrated that the relative expression of MAL2 mRNA and protein was markedly elevated in BC cells transfected with the METTL3 overexpression vector, supporting an association between METTL3 expression, m^6^A modification, and MAL2 abundance in BC cells (Fig. 5C and 5D). Importantly, APS treatment notably reduced the mRNA and protein levels of METTL3 in MCF-7 and MDA-MB-231 cells (Fig. 5E and 5F). To further clarify the relationship between APS treatment and METTL3-mediated regulation of MAL2, rescue experiments were performed by transfecting METTL3 overexpression vectors in APS-treated BC cells. qRT-PCR and western blotting analysis demonstrated that APS-induced downregulation of MAL2 mRNA and protein was partially reversed upon METTL3 overexpression in BC cells (Fig. 5G and 5H). Collectively, these results implied that APS might suppress MAL2 expression by modulation of METTL3-associated m^6^A regulatory mechanisms.

## Discussion

In recent years, APS has demonstrated antitumor effects, enhanced treatment outcomes while reducing toxicity and side effects in various cancer types, including BC [7, 25]. In this work, we systematically investigated the antitumor effects of APS and explored its potential regulatory mechanism in BC. It was observed that APS treatment markedly reduced the proliferation, migration and invasion of BC cells *in vitro* and restricted the tumor growth of BC cells xenograft *in vivo*. Innovatively, this study revealed that APS remarkably downregulated the MAL2 expression in BC cells, likely by reducing its m^6^A modification through the suppression of METTL3. Therefore, the METTL3/MAL2 axis exhibits potential as a therapeutic target for the enhancement of the anti-tumor effects of APS in BC.

*A. membranaceus* is a perennial herb that belong to the legume family and its dried roots are rich in various bioactive compounds, including polysaccharide fractions [26]. APS, a key component of polysaccharide fractions, has previously been employed in clinical trials for the treatment of gastric [27], colorectal [28], non-small-cell lung cancer [29], and pancreatic [30] cancers. Moreover, APS combined with adjuvant chemotherapy has shown to reduce chemotherapy-induced fatigue, insomnia, and improve overall health status, particularly in stage II/III premenopausal BC patients [31]. Previous research has disclosed that APS can hinder the proliferation, migration and invasion of BC cells *in vitro* [10, 32], and *in vivo* studies using triple-negative BC models have demonstrated reduced tumor growth following APS treatment [33]. Our observations were similar to these previous reports as we also found that APS treatment significantly reduced these malignant phenotypes of BC cells and restricted the tumor growth *in vivo*. However, beyond confirming its antitumor activity, our study provides new mechanistic insight by linking APS treatment to epitranscriptomic regulation in BC. While APS-associated modulation of m^6^A signaling has been described in other disease types [34-36], evidence for such regulation in BC has been lacking. Here, we for the first time identify MAL2 as a previously unrecognized m^6^A-regulated target in BC and demonstrate that APS downregulated MAL2 expression by reducing METTL3 expression to decrease m^6^A modification of MAL2. These findings distinguish the APS–METTL3–MAL2 axis from previously reported APS-related pathways and highlight a novel epitranscriptomic mechanism underlying the antitumor effects of APS in BC.

In the present study, APS was creatively found to result in a marked downregulation of MAL2 expression in BC. MAL2 has been reported to be overexpressed in BC, and its elevated levels have been associated with a worse prognosis [18]. Previous report suggests that MAL2 acts as a potential tumor promoter by modulating epithelial-mesenchymal transition (EMT), indicating its potential as a target for anti-metastatic therapy in BC [19, 37]. Interestingly, a study has closely linked the effects of APS to EMT [10], suggesting a potential convergence between APS and EMT-associated regulatory networks. While EMT is a key biological process driving cancer cell migration and invasion including BC [38], the upstream regulatory mechanisms linking APS to EMT modulation in BC remain poorly defined. Our findings identify MAL2 as a novel downstream target of APS and raise the possibility that APS-mediated suppression of EMT may involve MAL2 regulation, which has not been previously reported in the context of APS in BC. However, the precise contribution of MAL2 to APS-regulated EMT requires further experimental validation.

The m^6^A methylation regulates the functions of the target gene by influencing mRNA splicing, translation, degradation, and nuclear export, thereby controlling various biological processes [39]. To date, the m^6^A modification of MAL2 has not been implicated in the progression of BC. In addition, the role of m^6^A regulation in APS within the context of BC remains largely unstudied. In the present study, we provide the first evidence that APS treatment is associated with reduced METTL3 expression, accompanied by decreased m^6^A enrichment of MAL2 mRNA and downregulation of MAL2 expression, which together correlate with the suppression of BC progression. High METTL3 levels have been linked to BC tumorigenesis, progression, invasion, and metastasis by mediating m^6^A RNA modifications, which disrupt normal cell processes such as proliferation, migration, apoptosis, and the cell cycle [40]. Recently, a plant extract has been shown to reduce METTL3 expression and decrease RNA m^6^A modification of a target gene, but no such report exists for APS [24]. Therefore, our study uniquely identifies MAL2 as a downstream target linking APS-mediated METTL3 regulation to BC progression expand the potential epigenetic mechanism of APS in cancer.

In summary, our study provides the first evidence that APS inhibits the malignant properties of BC cells *in vitro* and *in vivo*, potentially through a METTL3-mediated m^6^A modification of MAL2. Decreased METTL3 expression coincides with lower m^6^A enrichment of MAL2 and MAL2 expression at mRNA and protein levels, suggesting that APS may modulate MAL2 expression through a METTL3-related m^6^A regulatory mechanism. The findings of our study highlight the relevance of METTL3 and m^6^A modifications in APS-treated BC and identify MAL2 as a novel downstream target of APS. Given the intricate nature of BC tumor pathogenesis, future research should focus on uncovering additional downstream regulatory molecules and signaling pathways to fully elucidate the molecular mechanisms underlying the anti-tumor effects of APS.

## Figures and Tables

**Table 1:** Primers used for qRT-PCR analysis in this study.

Name	Primer sequence (5’→3’')
CD24-Forward	GACTCAGGCCAAGAAACGTC
CD24-Reverse	CCTGTTTTTCCTTGCCACAT
CXCL9-Forward	AGGAGTGACTTGGAACTCCATT
CXCL9-Reverse	TGGGGACAAGATGAGAAAGG
TPD52-Forward	GCTGCTTTTTCGTCTGTTGGCT
TPD52-Reverse	TCAAATGATTTAAAAGTTGGGGAGTT
ESRP1-Forward	CAATATTGCCAAGGGAGGTG
ESRP1-Reverse	GTCCCCATGTGATGTTTGTG
MAL2-Forward	GTCCGTGACAGCGTTTTTCTT
MAL2-Reverse	AATTGAGGCTGCTACGTTTATGT
METTL3-Forward	ACCCTGACAGATGATGAGATGC
METTL3-Reverse	CGTTCATACCCCCAGAGGTTTAG
GAPDH-Forward	TTAAAAGCAGCCCTGGTGAC
GAPDH-Reverse	CTCTGCTCCTCCTGTTCGAC
